# Supplementary material for: JP-14: A Trace Amine-Associated Receptor 1 Agonist with Anti-Metabolic Disorder Potential
Source: Int J Mol Sci. 2025 Oct 15;26(20):10033. doi: 10.3390/ijms262010033 (PMC12563652; doi:10.3390/ijms262010033)
Supplement: Supplementary file 1 [file ijms-26-10033-s001.zip › ijms-3878725-supplementary.pdf]

Supporting Information for

Article

JP-14: A Trace Amine-Associated Receptor 1 Agonist with Anti-Metabolic Disorder Potential

Monika Marcinkowska [1], Joanna Sniecikowska [1], Monika Głuch-Lutwin [2], Barbara Mordyl [2], Marek Bednarski [3], Adam Bucki [1], Michał Sapa [1], Monika Kubacka [3], Agata Siwek [2], Agnieszka Zagórska [1], Jacek Sapa [3],
Marcin Kołaczkowski [1] and Magdalena Kotańska [3,*]

[1] Department of Medicinal Chemistry, Faculty of Pharmacy, Jagiellonian University Medical College,
9 Medyczna Street, 30-688 Krakow, Poland; monika.marcinkowska@uj.edu.pl (M.M.); joanna.sniecikowska@uj.edu.pl (J.S.); adam.bucki@uj.edu.pl (A.B.); michal.piotr.sapa@doctoral.uj.edu.pl (M.S.); agnieszka.zagorska@uj.edu.pl (A.Z.); marcin.kolaczkowski@uj.edu.pl (M.K.)
[2] Department of Pharmacobiology, Jagiellonian University Medical College, 9 Medyczna Street, 30-688 Krakow, Poland; monika.gluch-lutwin@uj.edu.pl (M.G.-L.); barbara.mordyl@uj.edu.pl (B.M.);
agat.siwek@uj.edu.pl (A.S.)
[3] Laboratory of Pharmacological Screening, Department of Pharmacodynamics, Jagiellonian University
Medical College, 9 Medyczna Street, 30-688 Krakow, Poland; marek.bednarski@uj.edu.pl (M.B.); monika.kubacka@uj.edu.pl (M.K.); jacek.sapa@uj.edu.pl (J.S.)
* Correspondence: magda.dudek@uj.edu.pl

Contents

1. *In vitro* screening for the all the synthetized compounds 2
2. Preliminary cytotoxicity assessment of JP14.......... 3
3. General Procedure for the Preparation of Final Compounds and Comprehensive Characterization Data (^{1}H NMR, ^{13}C NMR, and ^{19}F NMR spectra; LC/MS chromatograms; Figures S1–S33). 4

1. *In vitro* screening for the all the synthetized compounds

Table S1. Chemical structures of synthesized compounds along with their functional activities at TAAR1 and off-target receptors (5-HT_{2C}, α_1, $\alpha_{2A-B,}$-adrenergic receptors)

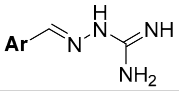

Compd	Ar	TAAR1 Ago EC_{50} [μM]	TAAR1 Ant EC_{50} [nM]	5-HT_2C Ago EC_{50} [nM]	5-HT_2C Ant EC_{50} [nM]	α_1 K_i [nM]	α_2 K_i [nM]	a_{2A} Ago EC_{50} [nM]	α_{2B} Ant EC_{50} [nM]
1 (JP01)	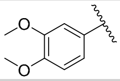	n.a.	n.a.	n.a.	n.a.	n.a.	n.a.	n.a.	n.a.
2 (JP02)	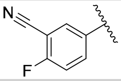	n.a.	n.a.	n.a.	n.a.	n.a.	995.5 ± 82.5	n.a.	n.a.
3 (JP03)	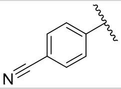	n.a.	n.a.	n.a.	n.a.	n.a.	n.a.	n.a.	n.a.
4 (JP06)	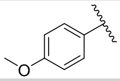	n.a.	n.a.	n.a.	n.a.	n.a.	1071.0 ± 78.0	n.a.	n.a.
5 (JP07)	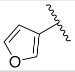	n.a.	n.a.	n.a.	n.a.	n.a.	n.a.	n.a.	n.a.
6 (JP08)	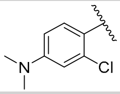	n.a.	n.a.	n.a.	n.a.	n.a.	n.a.	n.a.	n.a.
7 (JP09)	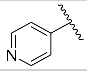	n.a.	n.a.	n.a.	n.a.	n.a.	n.a.	n.a.	n.a.
8 (JP10)	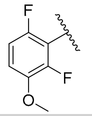	n.a.	n.a.	n.a.	n.a.	n.a.	231.5 ± 8.5	n.a.	220
9 (JP13)	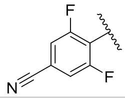	n.a.	n.a.	n.a.	n.a.	n.a.	n.a.	n.a.	n.a.
10 (JP14)	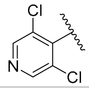	11.29 ± 1.4	n.a.	n.a.	n.a.	n.a.	126.0 ± 27.0	15%	622
GNB		0.301 ± 0.04 max Ago 40%	n.a.	n.a.	n.a.	n.t.	2.6 ±0.6	16.32	n.a.
4-OH GNB		34% max Ago (10^{-4}M)[a]	n.t.	n.t.	n.t.	n.t.	n.t.	316.3[a] Ago	330.2[a] Ant
TYR		5.87 ± 0.47	n.t.	n.t.	n.t.	n.t.	n.t.	n.t.	n.t.
RTI		n.a.	167.6 ± 50.7	n.t.	n.t.	n.t.	n.t.	n.t.	n.t.
5-HT		n.a.	n.a.	0.8 ± 0.1	n.t.	n.t.	n.t.	n.a.	n.t.
MSE		n.a.	n.a.	n.t.	1.1 ± 0.1	n.t.	n.t.	n.a.	231 i 126 nM
PHT		n.t.	n.t.	n.t.	n.t.	10.9 ± 0.8	n.t.	n.t.	n.t.
PHE		n.t.	n.t.	n.t.	n.t.	n.t.	159 ± 22.1	n.t.	n.t.
CLO		n.t.	n.t.	n.t.	n.t.	n.t.	3.1 ±0.4	n.t.	n.t.
BRIM		n.t.	n.t.	n.t.	n.t.	n.t.	n.t.	7.25	n.a.
YOH		n.t.	n.t.	n.t.	n.t.	n.t.	n.t.	n.a.	5.43 ± 1.8

n.a.- no activity at 10^{-5}M; n.t. - not tested; GNB – guanabenz; TYR – tyramine, RTI - RTI-7470-44; 5-HT – serotonin; MSE – methysergide; PHT – phentolamine; PHE – phenylephrine; CLO – clonidine; a – [7]

2. Preliminary cytotoxicity assessment of JP14

Table S2. Assessment of membrane integrity following exposure to tested compounds

Compound	Concentration of the tested compounds [μM]						IC_{50}	R2
		100	50	10	1	0.1	μM	
JP 14	X	8	10	6	6	6	N.C.	N.C.
	SD	5	8	4	4	4	N.C.	N.C.
TYRAMINE	X	11	8	7	7	8	N.C.	N.C.
	SD	5	7	5	7	5	N.C.	N.C.

Experiments performed using the **HEPG2 cell line**. Values are expressed as percentage relative to control (death cells); N.C. — not calculable; mean ± SD; n = 9; R2- coefficient of determination

Table S3. Assessment of membrane integrity following exposure to tested compounds

Compound	Concentration of the tested compounds [μM]						IC 50	R2
		100	50	10	1	0.1	μM	
JP 14	X	5	4	5	6	6	N.C.	N.C.
	SD	2	1	1	0	1	N.C.	N.C.
TYRAMINE	X	8	6	6	6	6	N.C.	N.C.
	SD	2	1	0	1	0	N.C.	N.C.

Experiments performed using the **3T3L1 cell line**. Values are expressed as percentage relative to control (death cells); N.C. — not calculable; mean ± SD; n = 9; R2- coefficient of determination

Table S4. Effect of test compounds on cellular metabolic function

Compound	Concentration of the tested compounds [μM]						IC 50	R2
		100	50	10	1	0.1	μM	
JP 14	X	60	71	95	101	96	N.C.	N.C.
	SD	3	3	4	8	11	N.C.	N.C.
	SD	5	7	7	8	16	N.C.	N.C.
TYRAMINE	X	80	89	97	98	101	N.C.	N.C.
	SD	5	10	14	9	11	N.C.	N.C.

Experiments performed using the **HEPG2 cell line**. Values are expressed as percentage relative to control (live cells). N.C. — not calculable; mean ± SD; n = 9; R2- coefficient of determination

Table S5. Effect of test compounds on cellular metabolic function

Compound		Concentration of the tested compounds [μM]					IC 50	R2
		100	50	10	1	0.1	μM	
JP 14	X	51	80	82	85	87	N.C.	N.C.
	SD	4	18	9	21	16	N.C.	N.C.
TYRAMINE	X	55	67	88	90	95	N.C.	N.C.
	SD	12	18	11	11	5	N.C.	N.C.

Experiments performed using the **3T3-L1 cell line**. Values are expressed as percentage relative to control (live cells); N.C. — not calculable; mean ± SD; n = 9; R2- coefficient of determination

3. General Procedure for the Preparation of Final Compounds and Comprehensive Characterization Data (^{1}H NMR, ^{13}C NMR, and ^{19}F NMR spectra; LC/MS chromatograms; Figures S1–S33).

a. General procedure:

The designed set of molecules was synthesized in a highly efficient and cost-effective one-step synthesis. The appropriate aldehyde (1 equiv.) and aminoguanidine hydrochloride (1.1 equiv.) were mixed with methanol (5 mL) and stirred at reflux for 2-3 hours. Then, the reaction mixture was cooled to room temperature, and diethyl ether and petroleum ether were added. The pure product was then crystallized from the reaction mixture. The obtained precipitate was washed with a 1:1 (v/v) mixture of diethyl ether and petroleum ether and then dried under reduced pressure. The final products were obtained in yields ranging from 41% to 98%.

b. Full characterization of the target compounds:

i. Characterizations of compound JP01

(E)-2-(3,4-dimethoxybenzylidene)hydrazine-1-carboximidamide

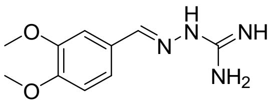

Formula: $C_{10}H_{14}N_4O_2$;

^{1}H NMR (500 MHz, DMSO-d_6) : δ 11.98 (br s, 1H), 8.04 (s, 1H), 7.99–7.56 (m, 3H), 7.53–7.45 (m, 1H), 7.24–7.19 (m, 1H), 6.96 (d, J = 8.3 Hz, 1H), 3.78 (s, 3H), 3.76 (s, 3H)

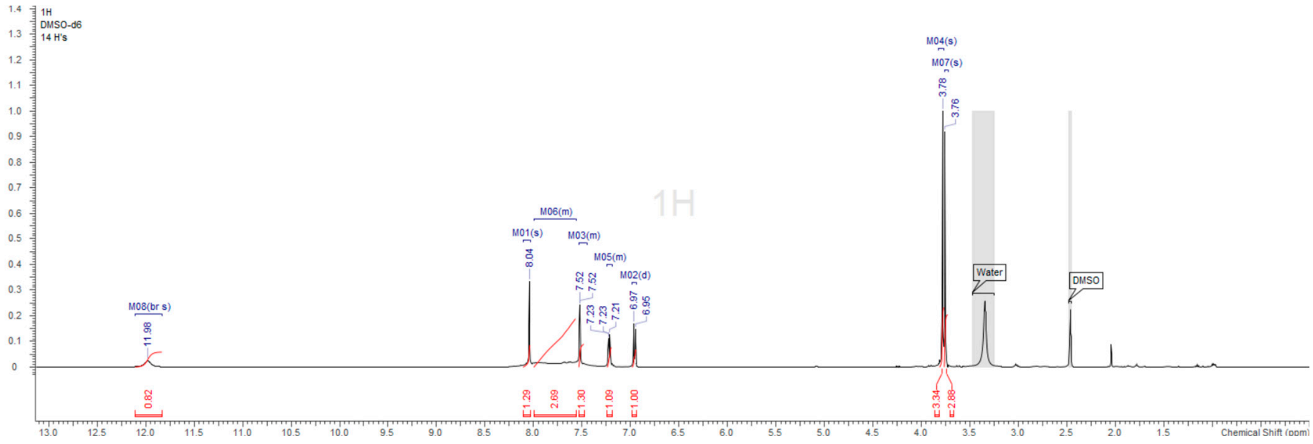

Figure S1. ^{1}H NMR (500 MHz, $(CD_3)_2SO$) spectrum of compound JP01

^{13}C NMR (126 MHz, DMSO-d_6): δ 155.85, 151.55, 149.60, 147.30, 126.66, 123.08, 111.72, 109.38, 56.24, 56.08

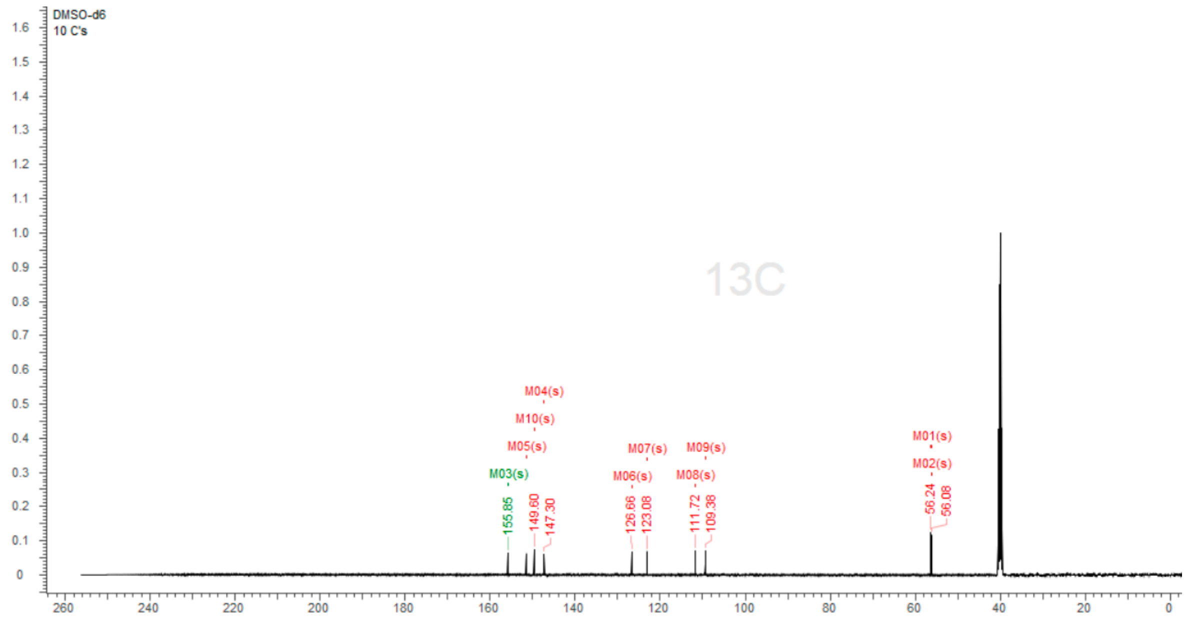

Figure S2. ^{13}C NMR (126 MHz, $(CD_3)_2SO$) spectrum of compound JP01

MS (ESI$^+$) *m/z*: 223.121 [M + H$^+$]

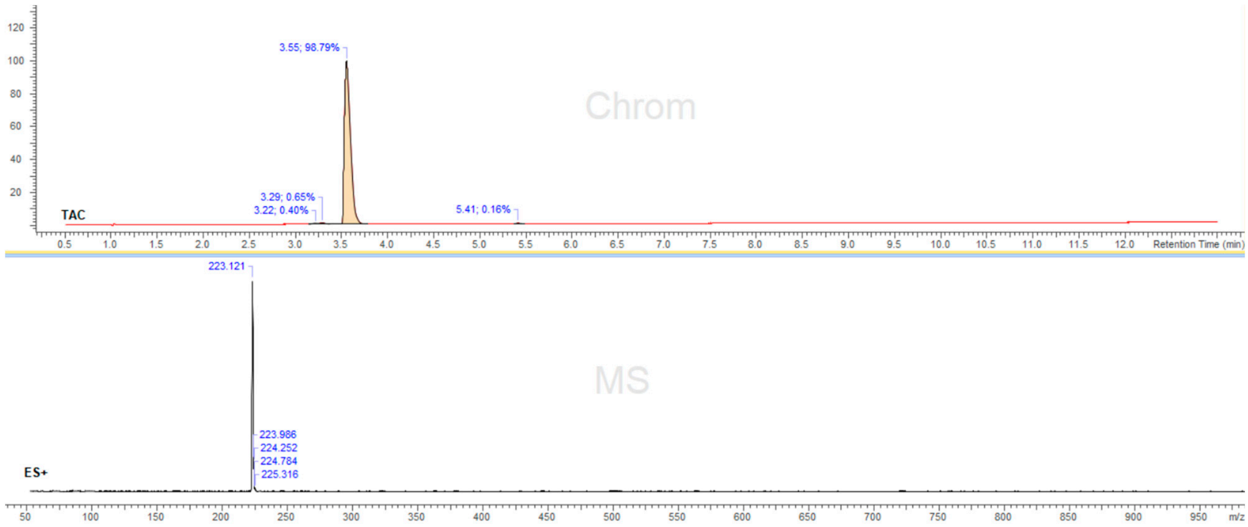

Figure S3. LC-MS chromatograms of compound JP01 (99.34%)

ii. Characterizations of compound JP02

(E)-2-(3-cyano-4-fluorobenzylidene)hydrazine-1-carboximidamide

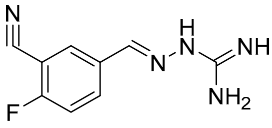

Formula: $C_9H_8FN_5$

^{1}H NMR (500 MHz, DMSO-d_6): δ 12.22 (br s, 1H), 8.54 (dd, J = 2.0, 6.3 Hz, 1H), 8.22–8.18 (m, 1H), 8.16 (s, 1H), 8.14–7.63 (m, 3H), 7.59 (t, J = 9.0 Hz, 1H)

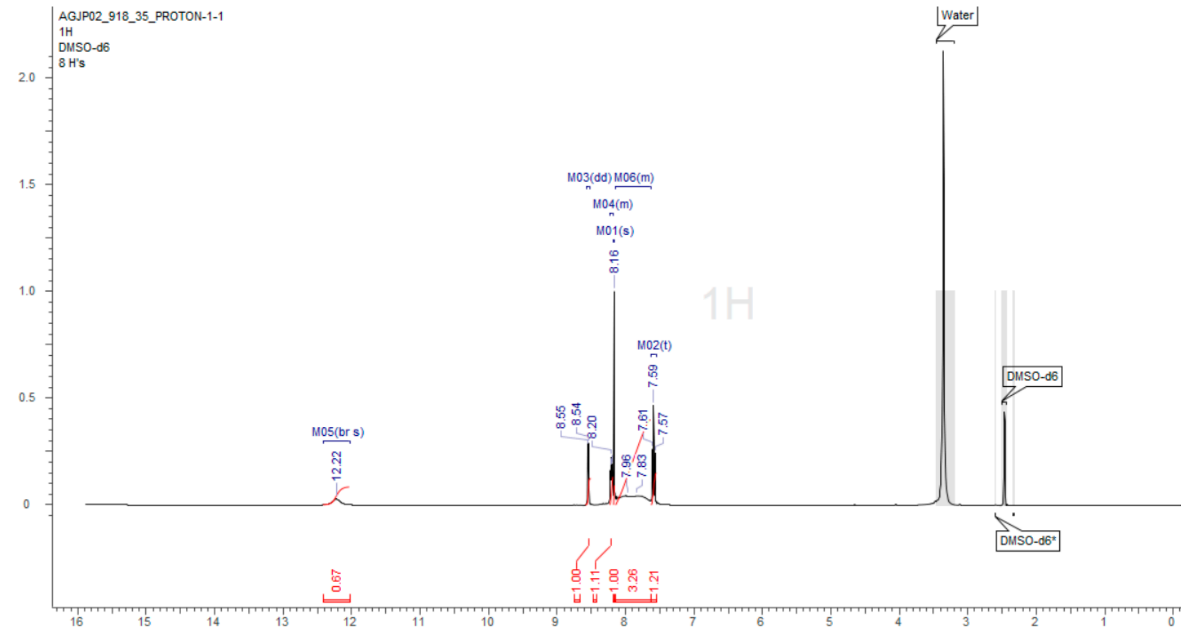

Figure S4. ^{1}H NMR (500 MHz, $(CD_3)_2SO$) spectrum of compound JP02

^{13}C NMR (126 MHz, DMSO-d_6): δ 163.50 (d, J = 259.1 Hz), 156.06, 143.94, 135.75 (d, J = 9.0 Hz), 132.96, 131.89, 117.57 (d, J = 20.1 Hz), 114.31, 101.42 (d, J = 16.2 Hz)

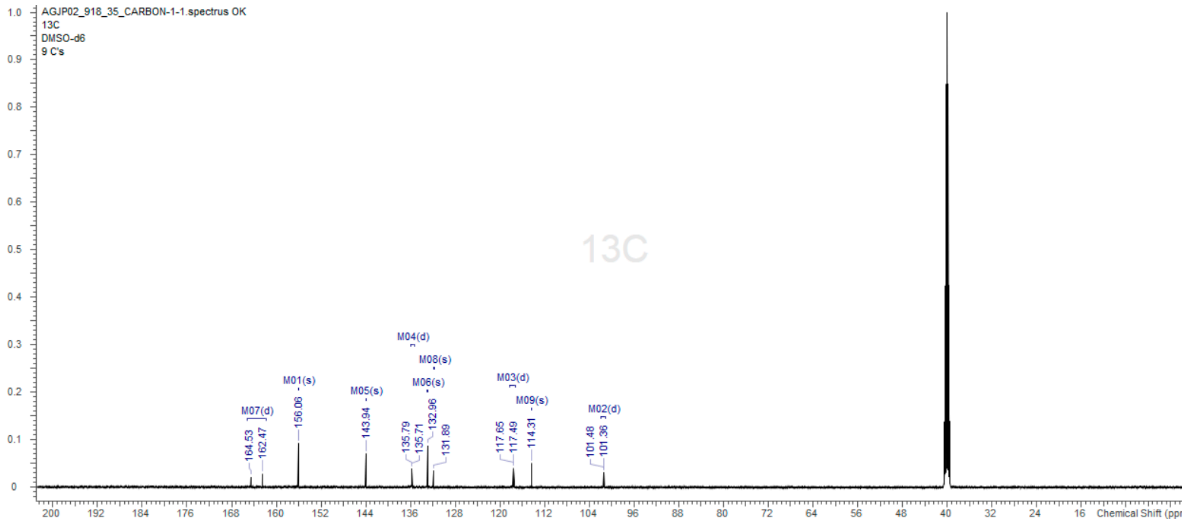

Figure S5. ^{13}C NMR (126 MHz, $(CD_3)_2SO$) spectrum of compound JP02

MS (ESI^+) *m/z*: 206.022 [$M + H^+$]

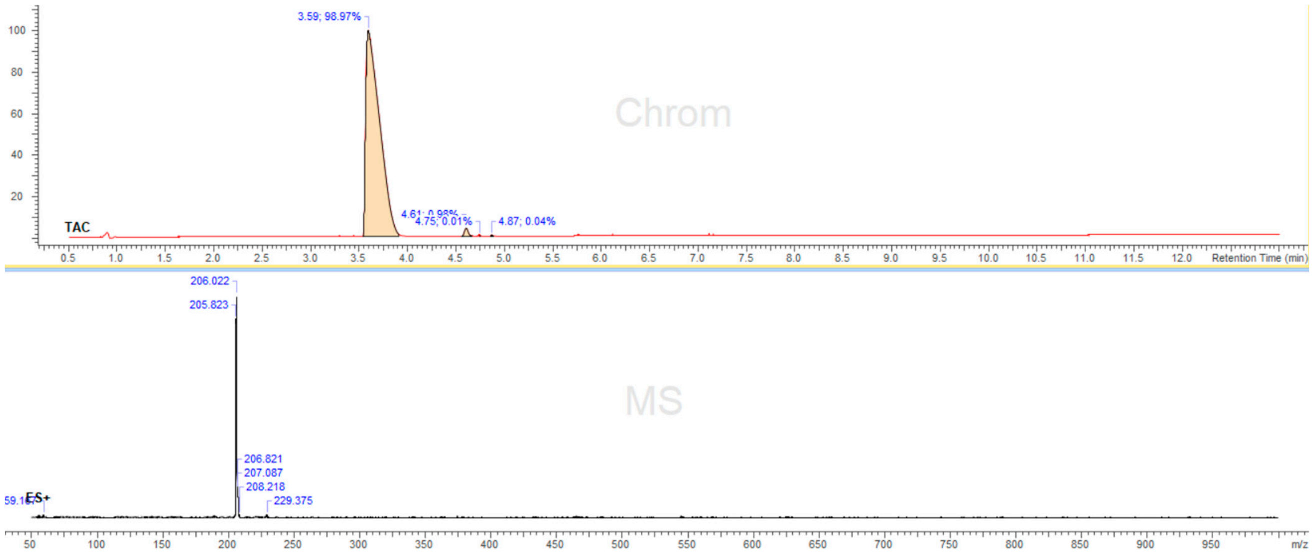

Figure S6. LC-MS chromatograms of compound JP02 (98.97%)

iii. Characterizations of compound JP03

(E)-2-(4-cyanobenzylidene)hydrazine-1-carboximidamide

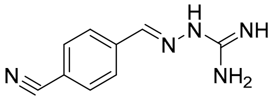

Formula: $C_9H_9N_5$

^{1}H NMR (500 MHz, DMSO-*d_6*): δ 12.29 (br s, 1H), 8.21 (s, 1H), 8.04 (d, *J* = 8.5 Hz, 2H), 7.88 (d, *J* = 8.3 Hz, 2H), 8.10 – 7.78 (m, 3H, overlapped with aromatic signals).

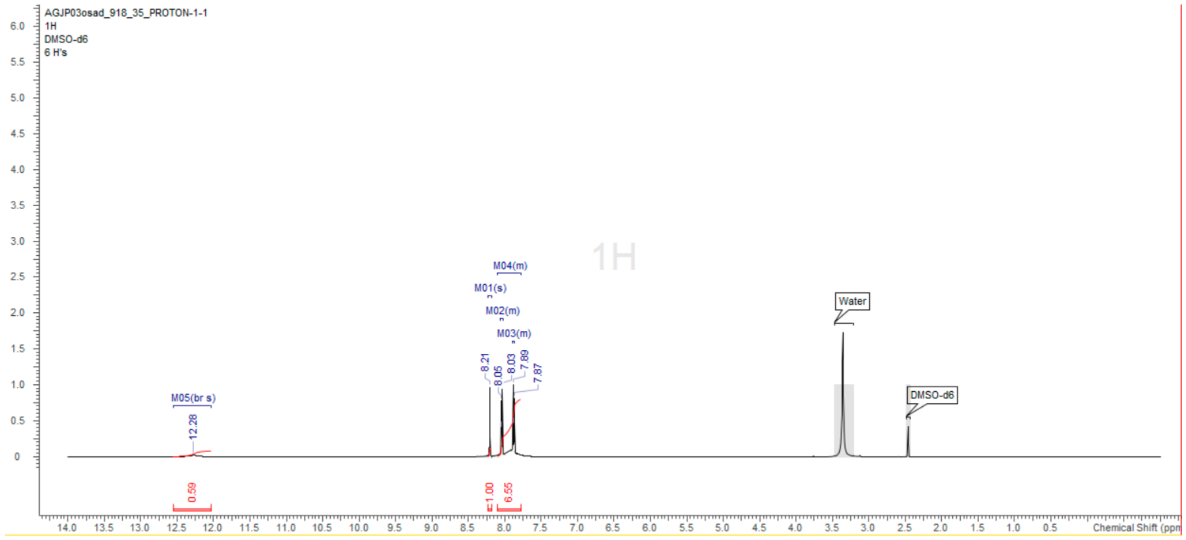

Figure S7. ^{1}H NMR (500 MHz, $(CD_3)_2SO$) spectrum of compound JP03

^{13}C NMR (126 MHz, DMSO-*d_6*): δ 156.04, 145.39, 138.43, 133.11 (2C), 128.68 (2C), 119.19, 112.76

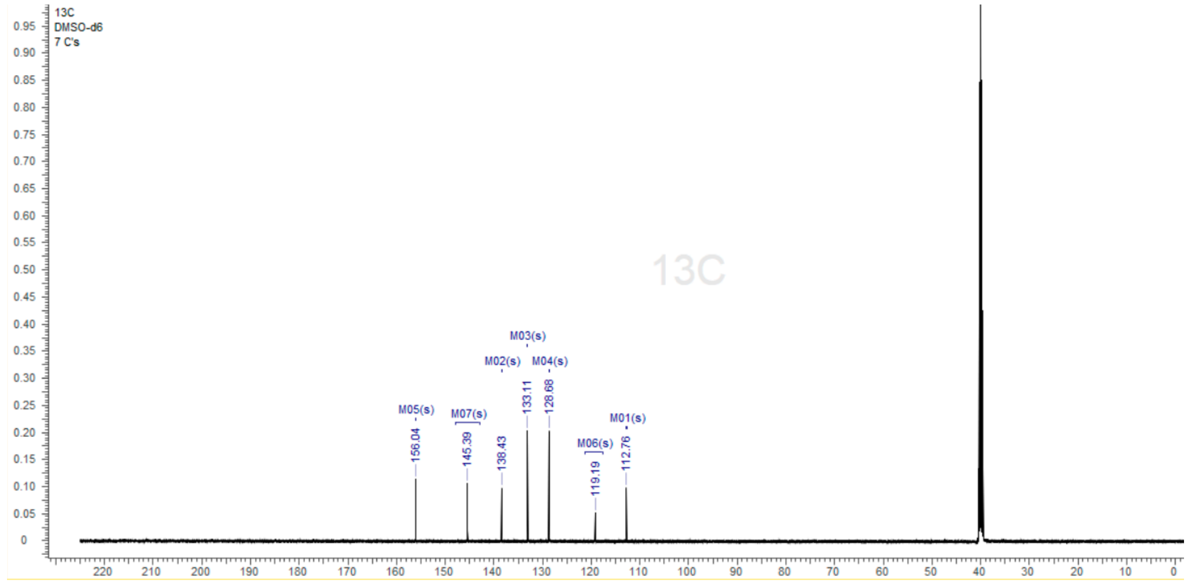

Figure S8. ^{13}C NMR (126 MHz, $(CD_3)_2SO$) spectrum of compound JP03

MS (ESI$^+$) *m*/*z*: 188.058 [M + H$^+$]

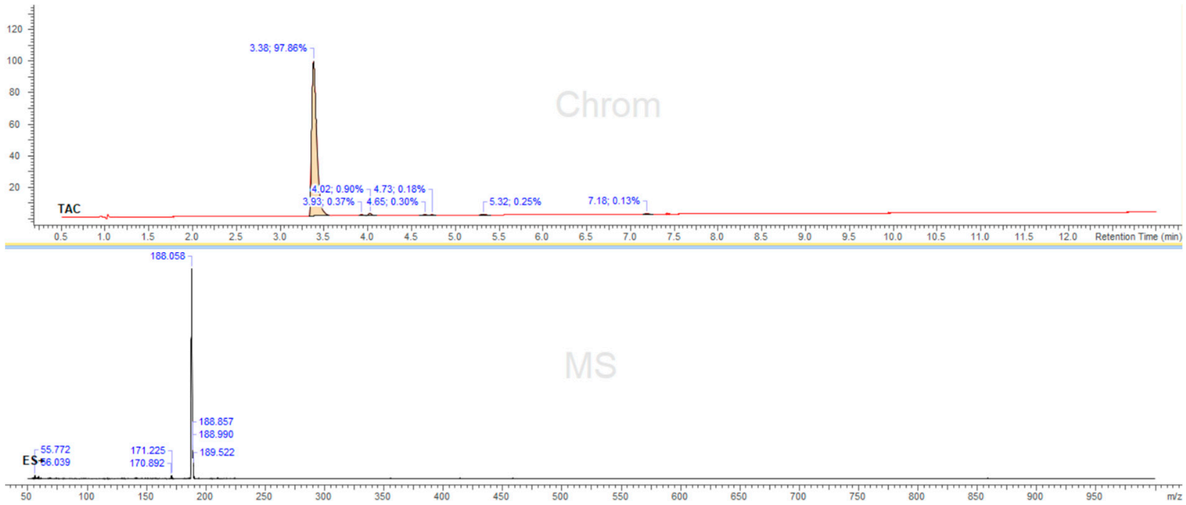

Figure S9. LC-MS chromatograms of compound JP03 (97.86%)

iv. Characterizations of compound JP06

(E)-2-(4-methoxybenzylidene)hydrazine-1-carboximidamide

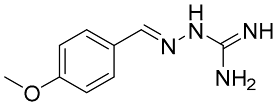

Formula: $C_9H_{12}N_4O$

^{1}H NMR (500 MHz, DMSO-d_6): δ 11.96 (s, 1H), 8.12 (s, 1H), 7.90-7.51 (br s, 3H overlaped with aromatic signals), 7.80 (d, J = 8.7 Hz, 2H), 7.00 (d, J = 8.9 Hz, 2H), 3.80 (s, 3H).

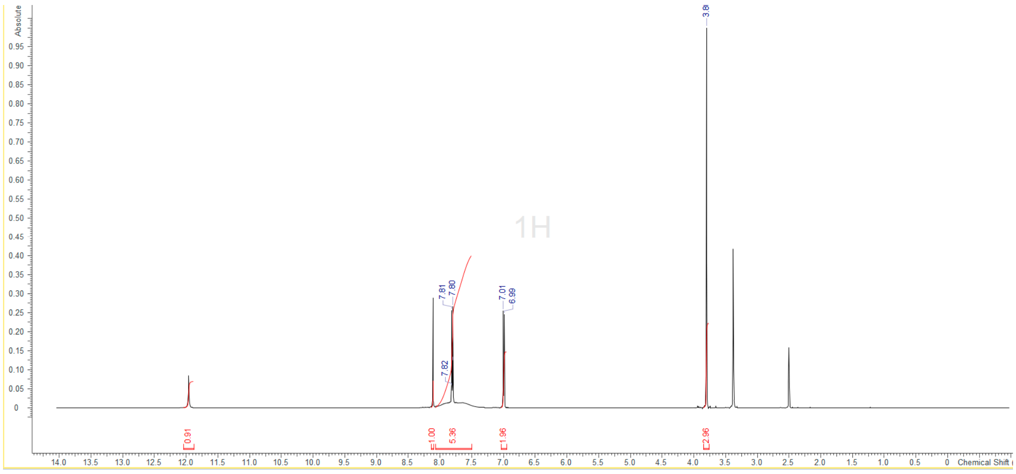

Figure S10. ^{1}H NMR (500 MHz, $(CD_3)_2SO$) spectrum of compound JP06

^{13}C NMR (126 MHz, DMSO-d_6): δ 161.30, 155.52, 146.72, 129.45, 126.20, 114.36, 55.53

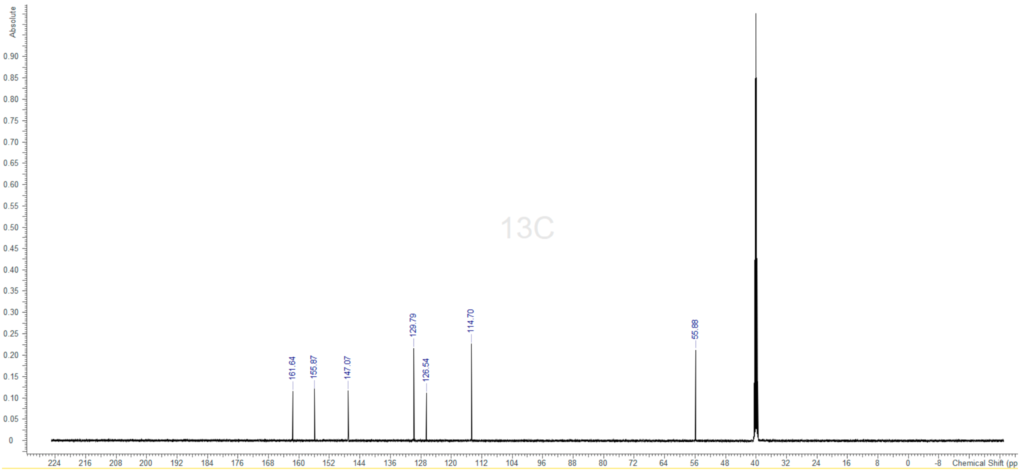

Figure S11. ^{13}C NMR (126 MHz, $(CD_3)_2SO$) spectrum of compound JP06

MS (ESI$^+$) *m/z*: 192.982 [M + H$^+$]

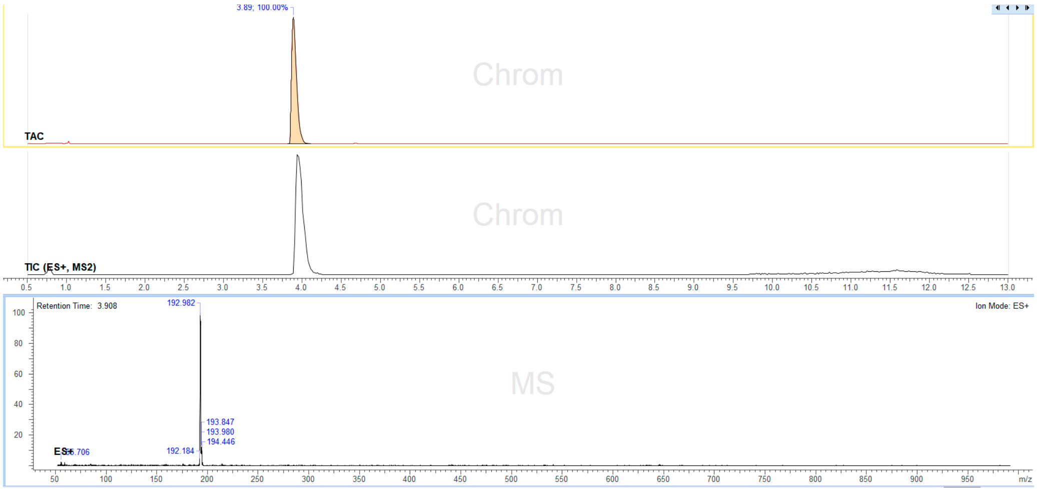

Figure S12. LC-MS chromatograms of compound JP06 (100.00%)

v. Characterizations of compound JP07

(E)-2-(furan-3-ylmethylene)hydrazine-1-carboximidamide

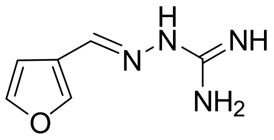

Formula: $C_6H_8N_4O$

^{1}H NMR (500 MHz, DMSO-*d*$_6$): δ 12.00 (br s, 1H), 8.13 (s, 1H), 8.11 (s, 1H), 7.71 (t, *J* = 1.6 Hz, 1H), 7.69 (br s, 3H, overlapped with aromatic signal), 6.98 (d, *J* = 1.7 Hz, 1H).

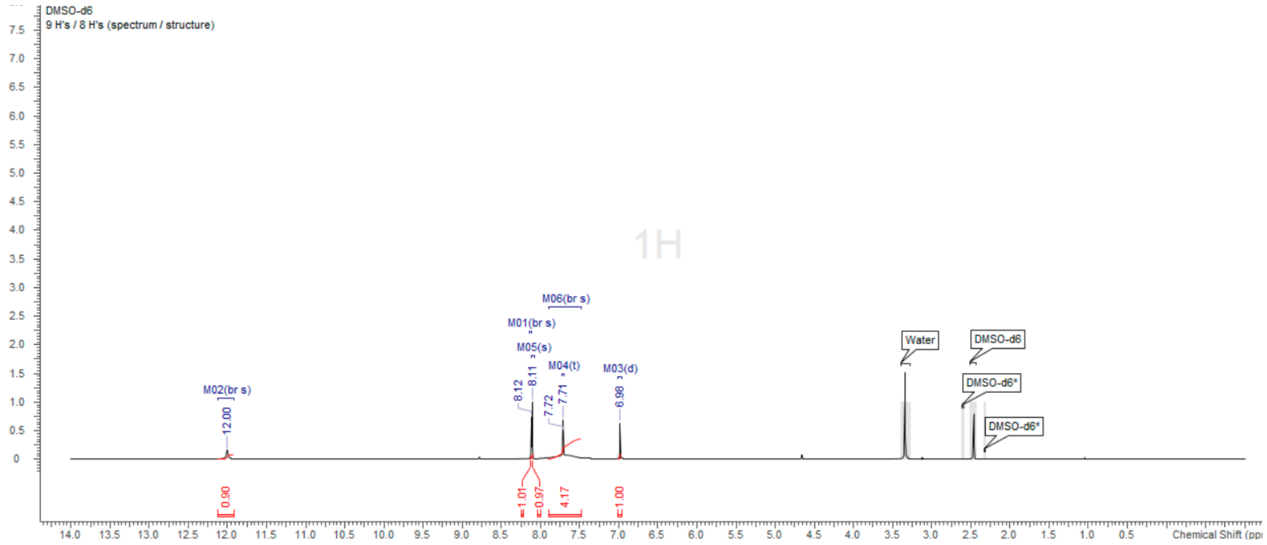

Figure S13. ^{1}H NMR (500 MHz, $(CD_3)_2SO$) spectrum of compound JP07

^{13}C NMR (500 MHz, DMSO-*d*$_6$): δ 155.85, 146.30, 145.18, 140.02, 122.63, 108.04

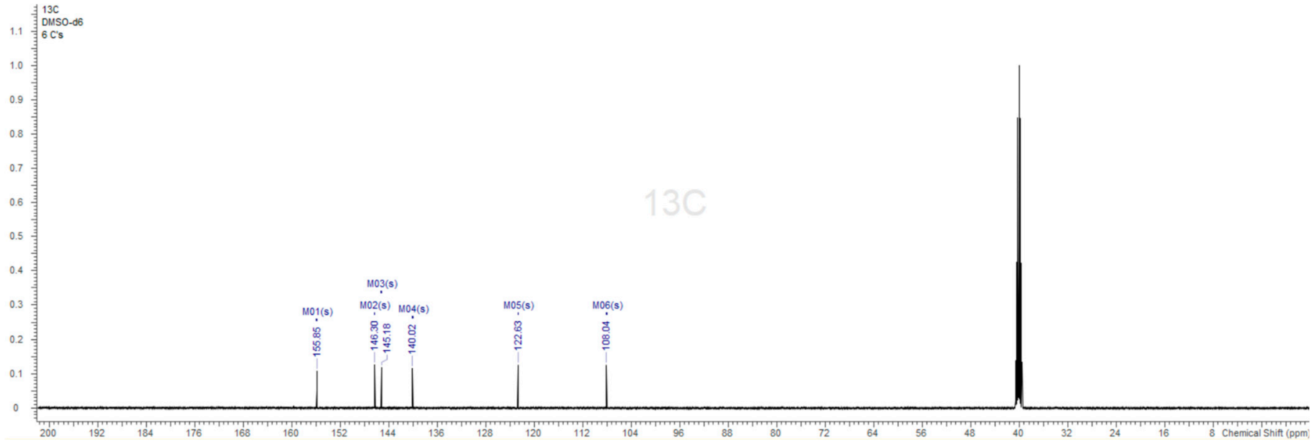

Figure S14. ^{13}C NMR (126 MHz, $(CD_3)_2SO$) spectrum of compound JP07

MS (ESI$^+$) *m*/*z*: 153.392 [M + H$^+$].

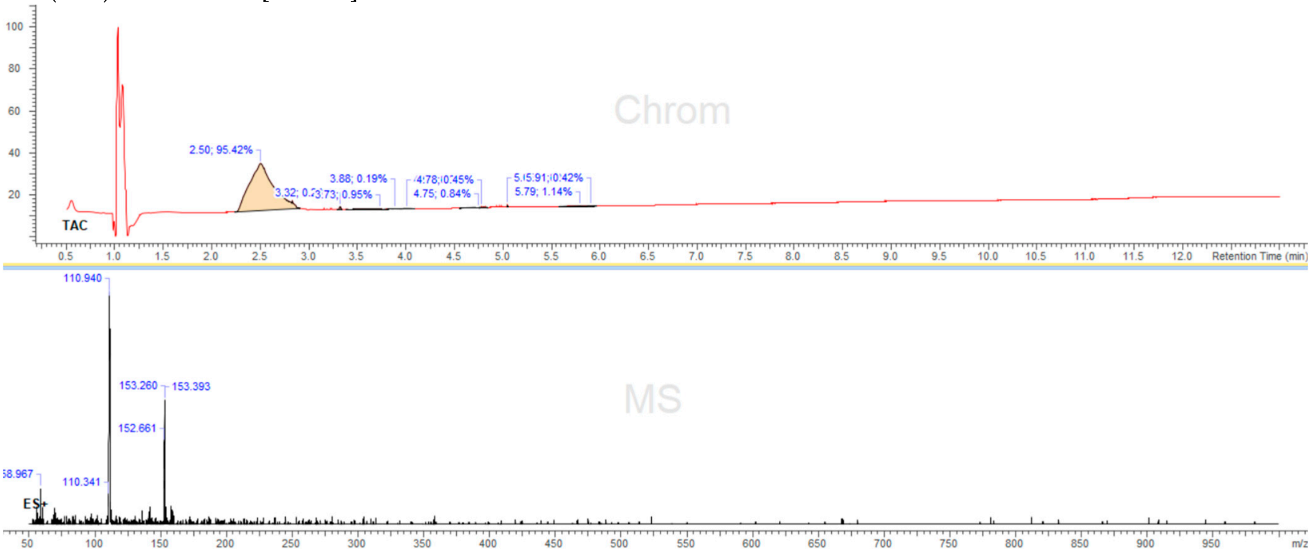

Figure S15. LC-MS chromatograms of compound JP07 (95.42%)

vi. Characterizations of compound JP08

(E)-2-(2-chloro-4-(dimethylamino)benzylidene)hydrazine-1-carboximidamide

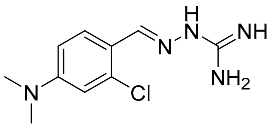

Formula: $C_{10}H_{14}ClN_5$

^{1}H NMR (500 MHz, DMSO-*d*$_6$): δ 11.94 (s, 1H), 8.38 (s, 1H), 8.13–8.00 (m, 1H), 7.71 (br s, 3H), 6.75–6.66 (m, 2H), 2.99 (s, 6H).

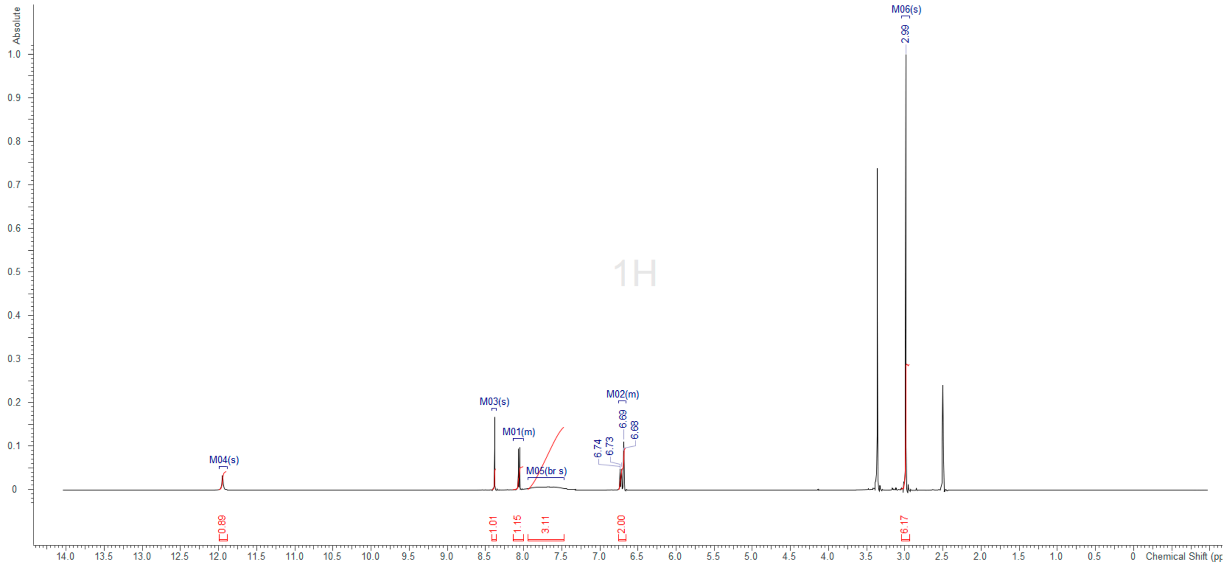

Figure S16. ^{1}H NMR (500 MHz, $(CD_3)_2SO$) spectrum of compound JP08

^{13}C NMR (126 MHz, DMSO-*d*$_6$): δ 155.02, 152.24, 143.47, 134.84, 128.28, 117.08, 111.15, 111.00.

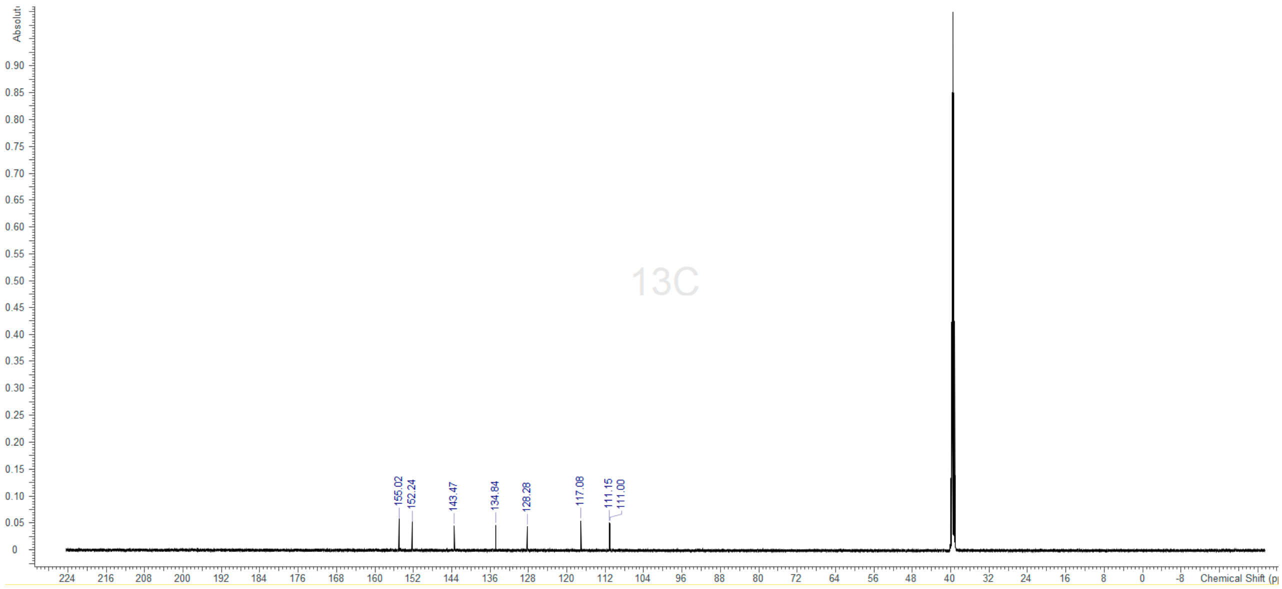

Figure S17. ^{13}C NMR (126 MHz, $(CD_3)_2SO$) spectrum of compound JP08

MS (ESI$^+$) *m/z*: 240.219 [M + H$^+$]

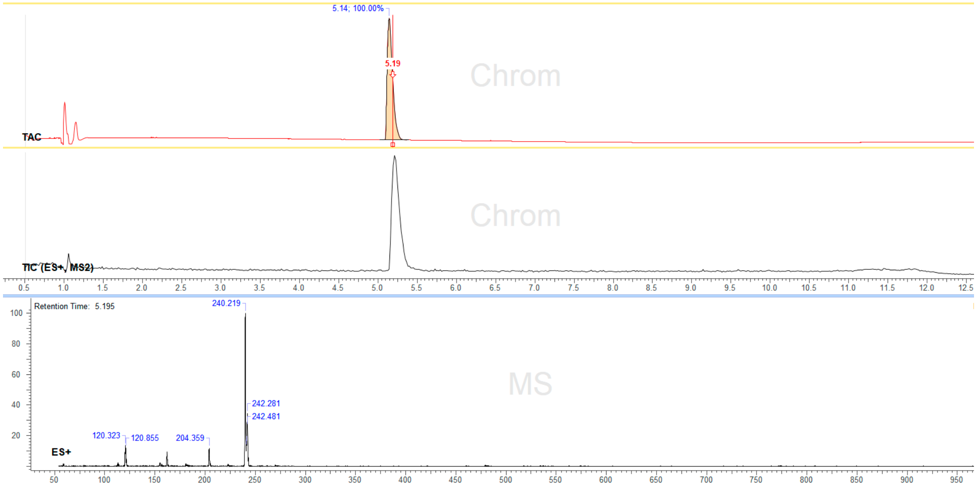

Figure S18. LC-MS chromatograms of compound JP08 (100.00%)

vii. Characterizations of compound JP09

E)-2-(pyridin-4-ylmethylene)hydrazine-1-carboximidamide

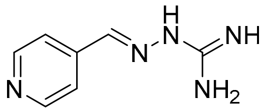

Formula: $C_7H_9N_5$

^{1}H NMR (500 MHz, DMSO-d_6): δ 12.43 (s, 1H), 8.74–8.54 (m, 2H), 8.20 (s, 1H), 8.11–7.86 (m, 3H), 7.88–7.76 (m, 2H)

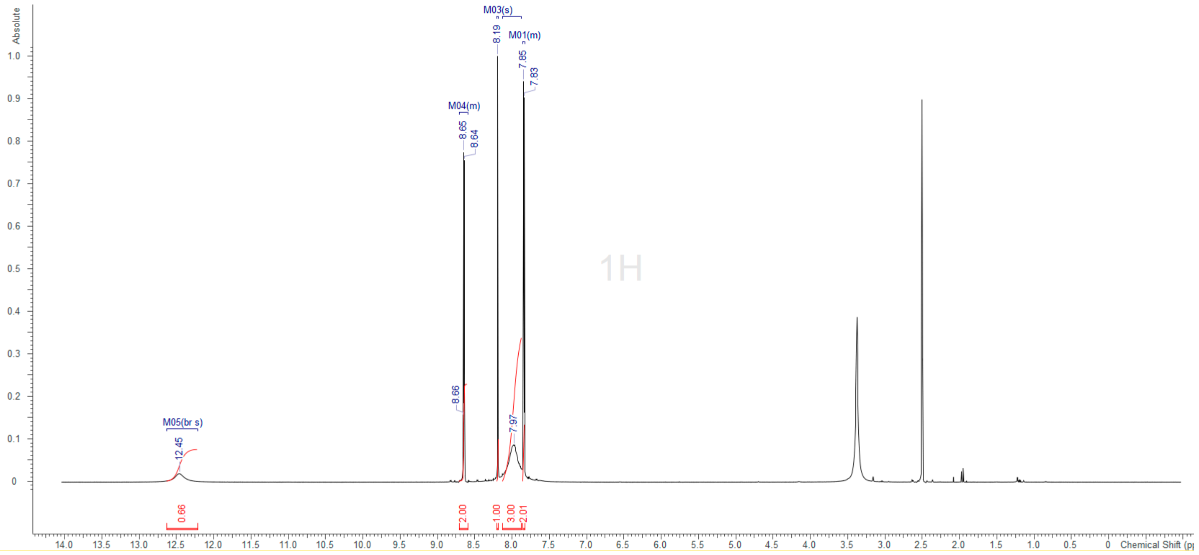

Figure S19. ^{1}H NMR (500 MHz, $(CD_3)_2SO$) spectrum of compound JP09

^{13}C NMR (126 MHz, DMSO-d_6): δ 156.17, 156.17, 150.66, 144.82, 141.20, 121.86, 40.28

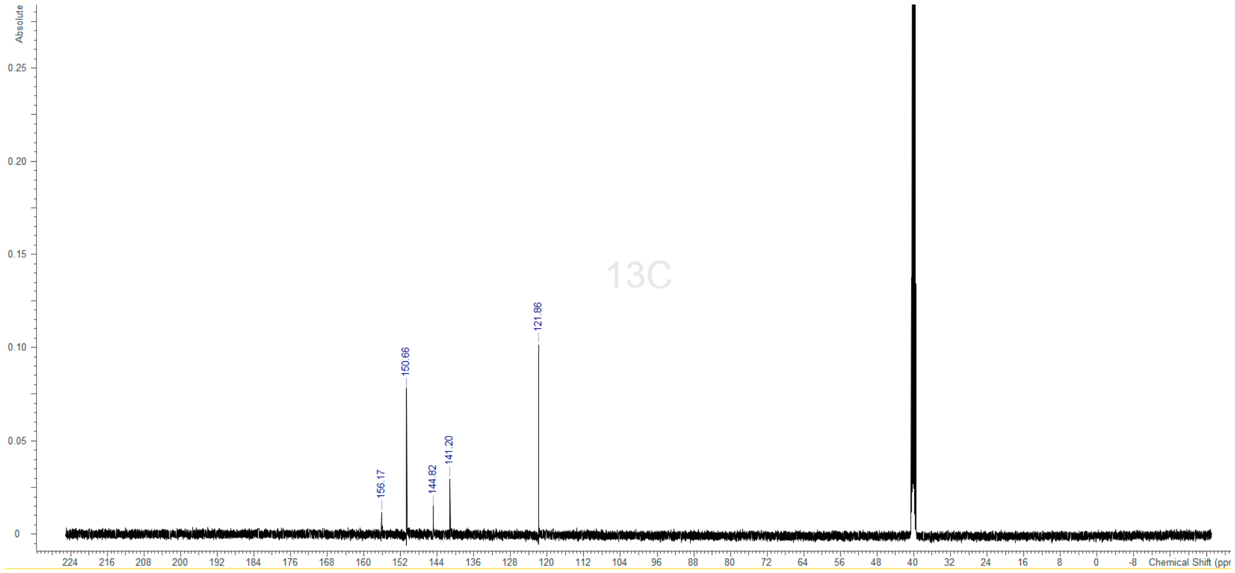

Figure S20. ^{13}C NMR (126 MHz, $(CD_3)_2SO$) spectrum of compound JP09

MS (ESI$^+$) *m/z*: 164.172 [M + H$^+$]

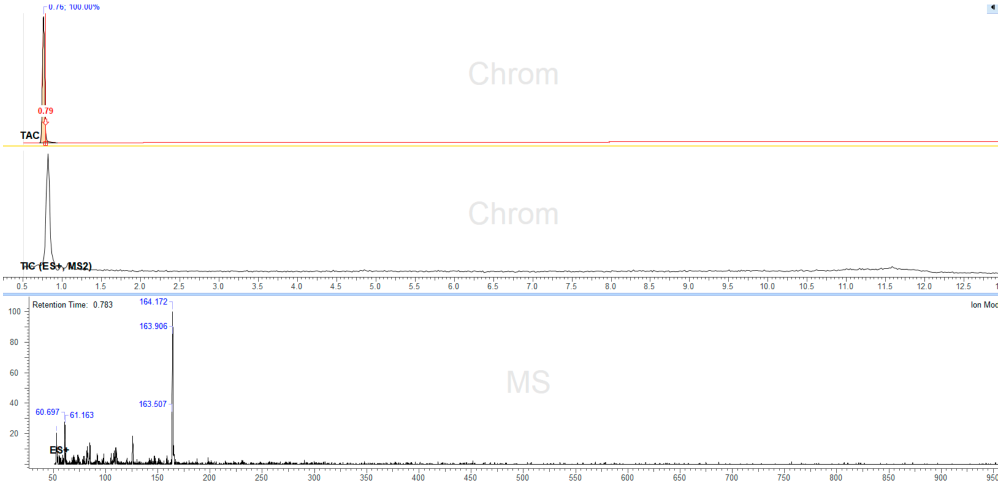

Figure S21. LC-MS spectra of compound JP09 (100.00%)

viii. Characterizations of compound JP10

(E)-2-(2,6-difluoro-3-methoxybenzylidene)hydrazine-1-carboximidamide

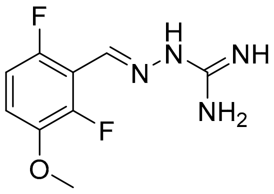

Formula: $C_9H_{10}F_2N_4O$

^{1}H NMR (500 MHz, CD_3OD): δ 8.28 (s, 1H), 7.20 (dt, *J* = 5.0, 9.2 Hz, 1H), 7.04–6.95 (m, 1H), 3.88 (s, 3H) N*H* protons not detected

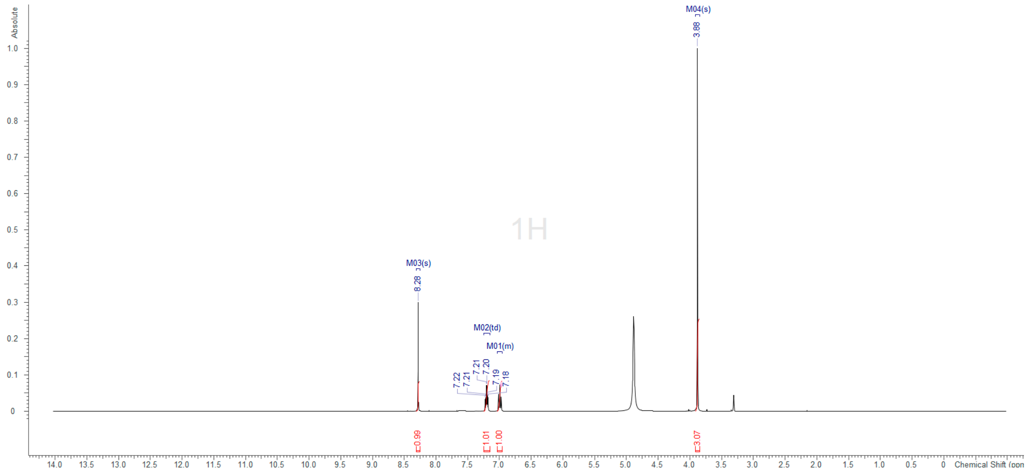

Figure S22. ^{1}H NMR (500 MHz, CD_3OD) spectrum of compound JP10

^{13}C NMR (126 MHz, CD_3OD): δ 156.83, 155.46, 151.49, 145.91, 139.65, 116.62, 112.26, 111.71, 57.18.

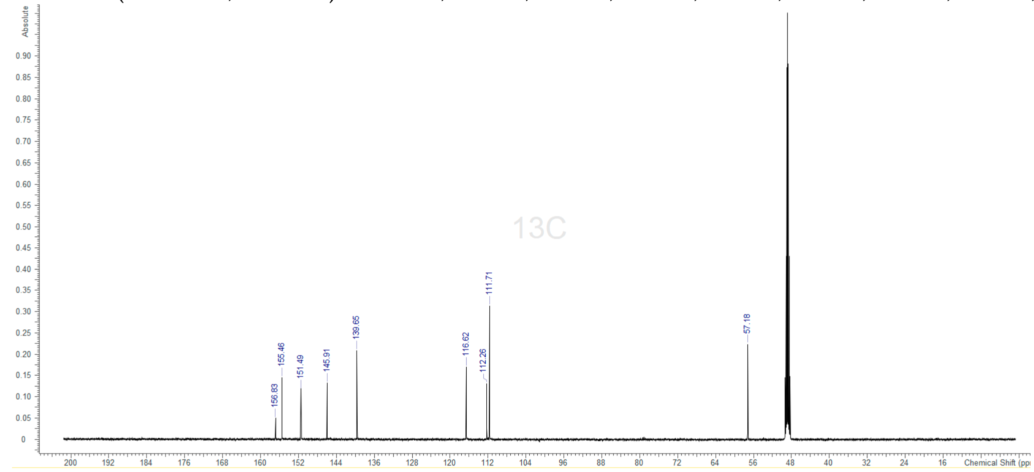

Figure S23. ^{13}C NMR (126 MHz, CD_3OD) spectrum of compound JP10

^{19}F NMR (471 MHz, CD_3OD): δ -122.96–-126.19 (m, 1F), -134.26 (dd, *J* = 1.5, 9.2 Hz, 1F)

MS (ESI$^+$) *m*/*z*: 229.109 [M + H$^+$]

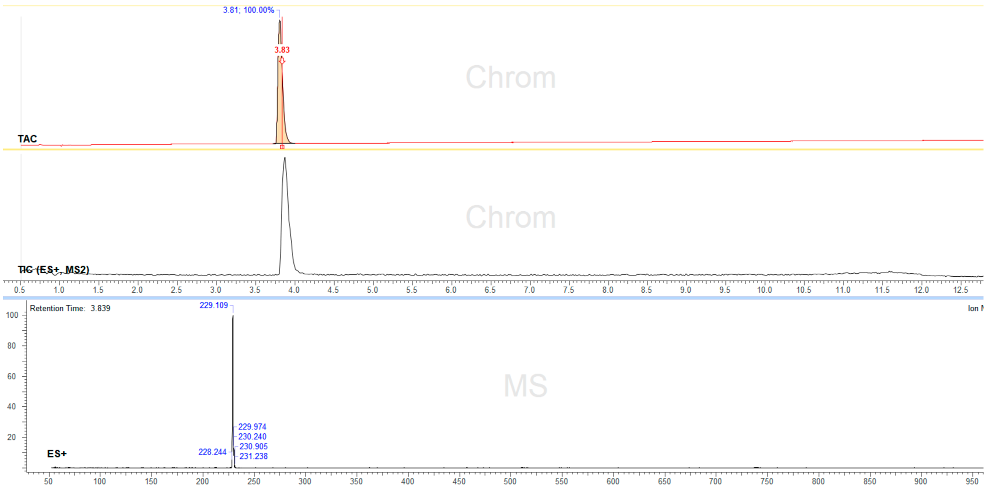

Figure S24. LC-MS chromatograms of compound JP10 (100.00%)

ix. Characterizations of compound JP13

(E)-2-(4-cyano-2,6-difluorobenzylidene)hydrazine-1-carboximidamide

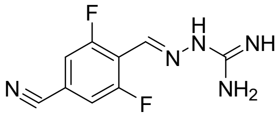

Formula: $C_9H_7F_2N_5$

^{1}H NMR (500 MHz, DMSO-d_6): δ 12.62 (br s, 1H), 8.30 (s, 1H), 7.90–7.87 (m, 2H), 7.96–7.73 (m, 3H)

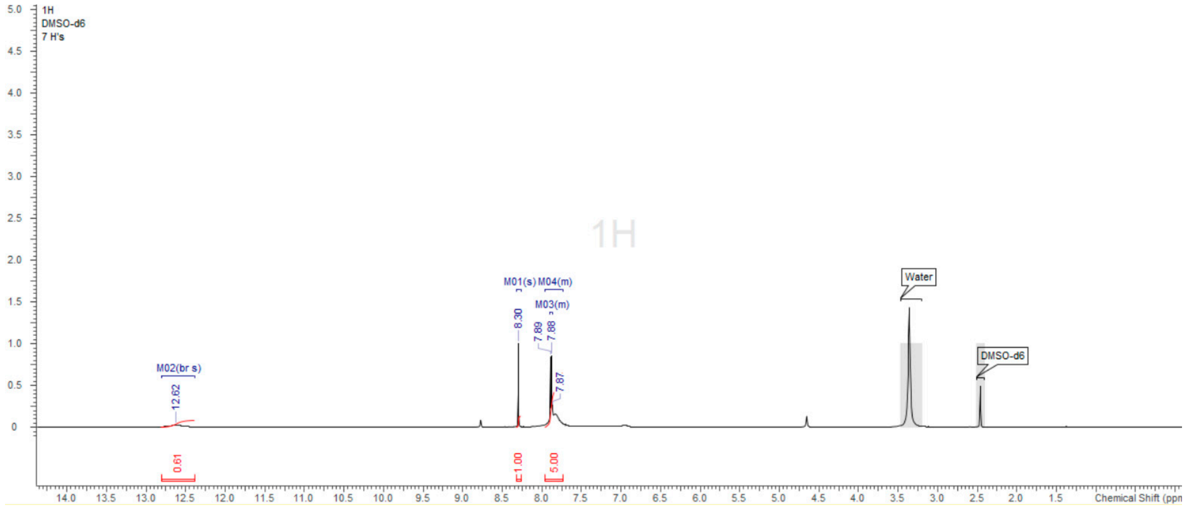

Figure S25. ^{1}H NMR (500 MHz, $(CD_3)_2SO$) spectrum of compound JP13

^{13}C NMR (126 MHz, DMSO-d_6, single pulse quintuple): δ 160.40, 155.84, 136.63, 117.35, 117.05, 116.59, 113.88

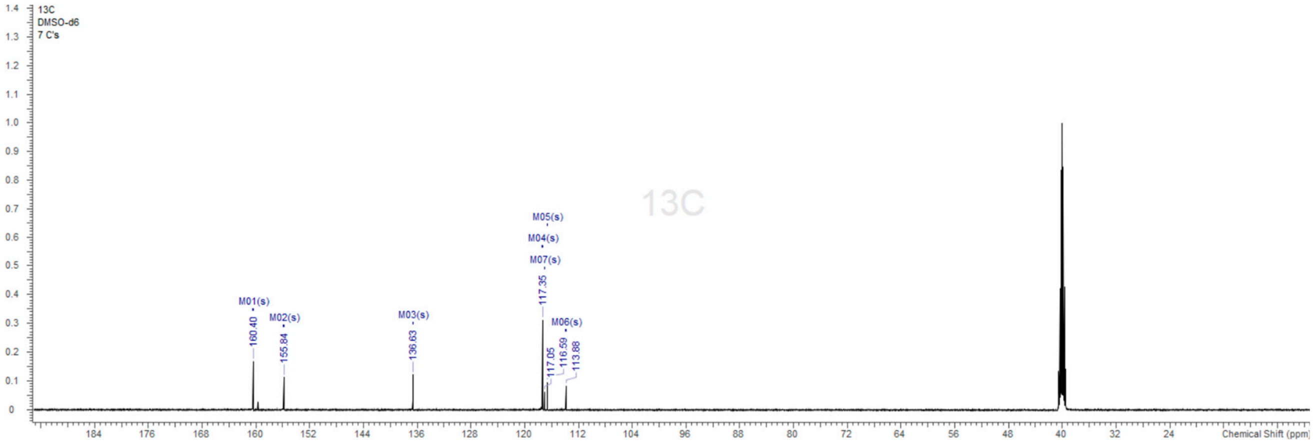

Figure S26. ^{13}C NMR (126 MHz, $(CD_3)_2SO$) spectrum of compound JP13 (single pulse quintuple)

^{13}C NMR (126 MHz, DMSO-*d*$_6$): δ 160.44 (br d, *J* = 257.3 Hz), 160.38 (br d, *J* = 257.3 Hz), 155.79, 136.68, 117.44 (d, *J* = 6.6 Hz), 117.26 (d, *J* = 6.6 Hz), 117.05 (br t, *J* = 3.1 Hz), 116.59 (t, *J* = 14.0 Hz), 113.89 (t, *J* = 13.0 Hz)

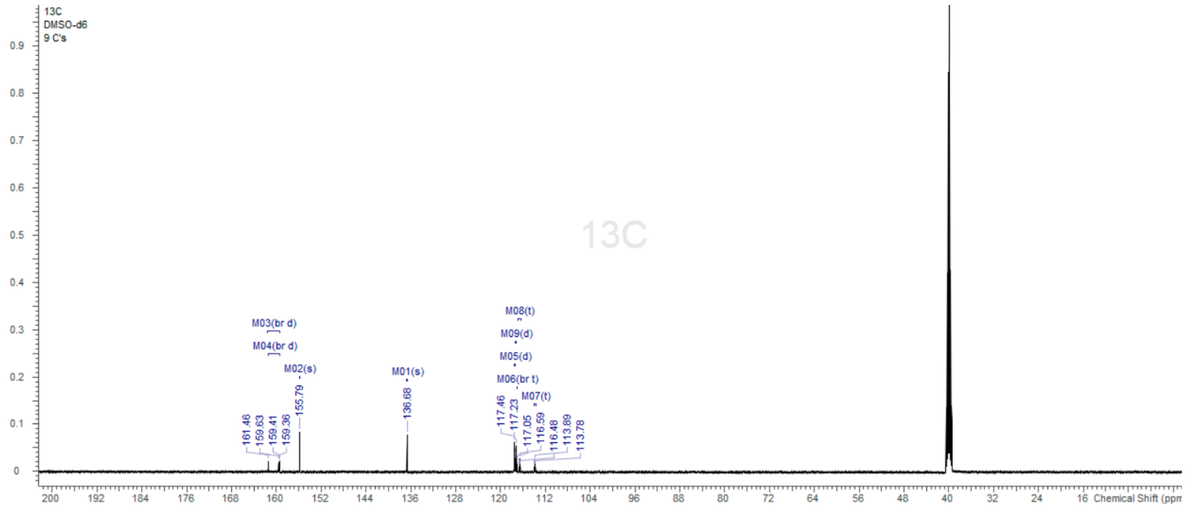

Figure S27. ^{13}C NMR (126 MHz, $(CD_3)_2SO$) spectrum of compound JP13

^{19}F NMR (471 MHz, DMSO-*d*$_6$): δ -108.79 (s, 2F)

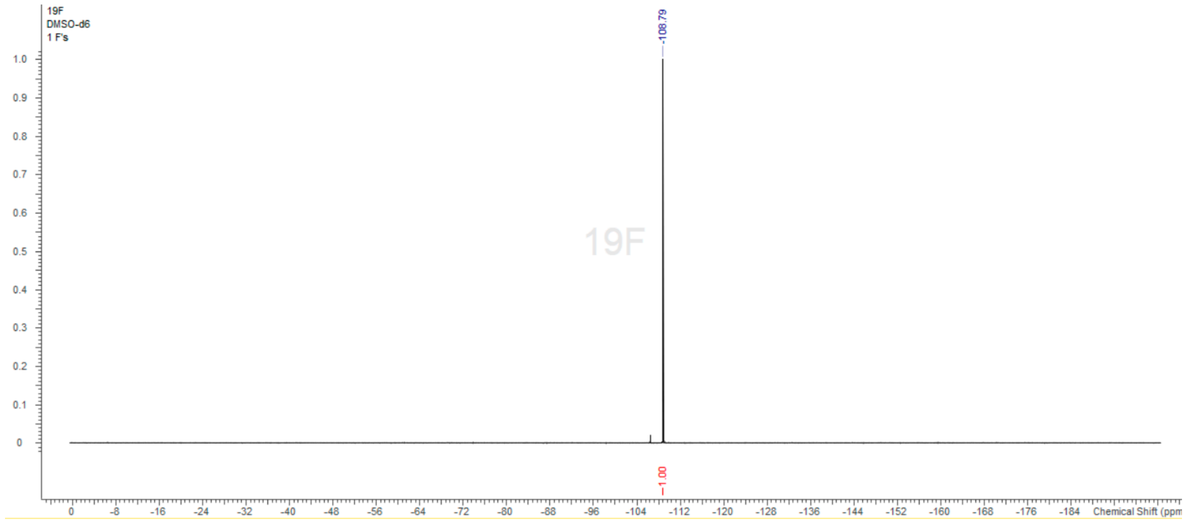

Figure S28. ^{19}F NMR (471 MHz, $(CD_3)_2SO$) spectrum of compound JP13

MS (ESI$^+$) *m*/*z*: 224.185 [M + H$^+$]

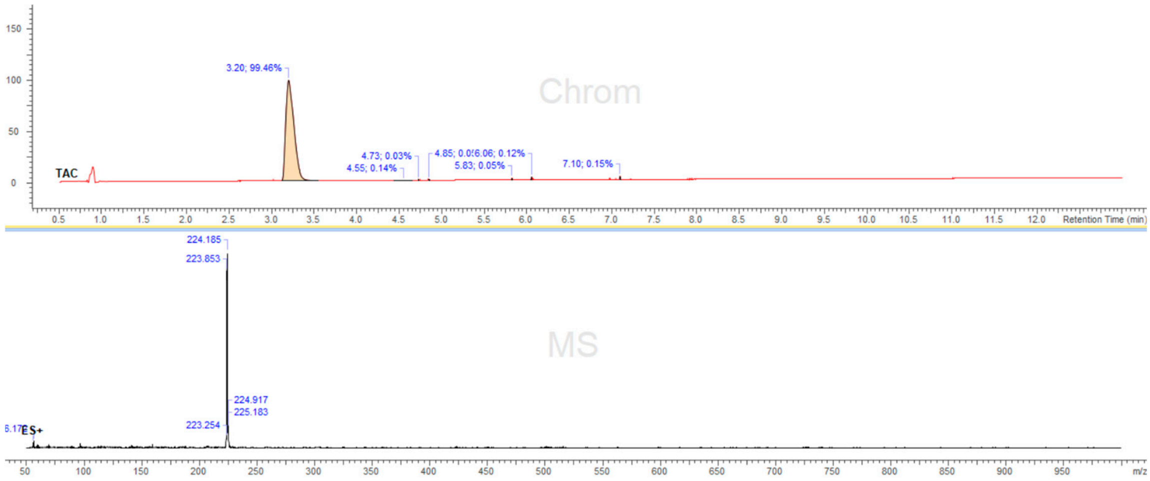

Figure S29. LC-MS chromatograms of compound JP13 (99.46%)

x. Characterizations of compound JP14

(E)-2-((3,5-dichloropyridin-4-yl)methylene)hydrazine-1-carboximidamide

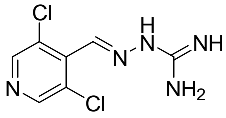

Formula: $C_7H_7Cl_2N_5$

^{1}H NMR (500 MHz, DMSO-*d*$_6$): δ 8.69 (s, 2H), 8.41 (s, 1H), 7.84 (br s, 4H).

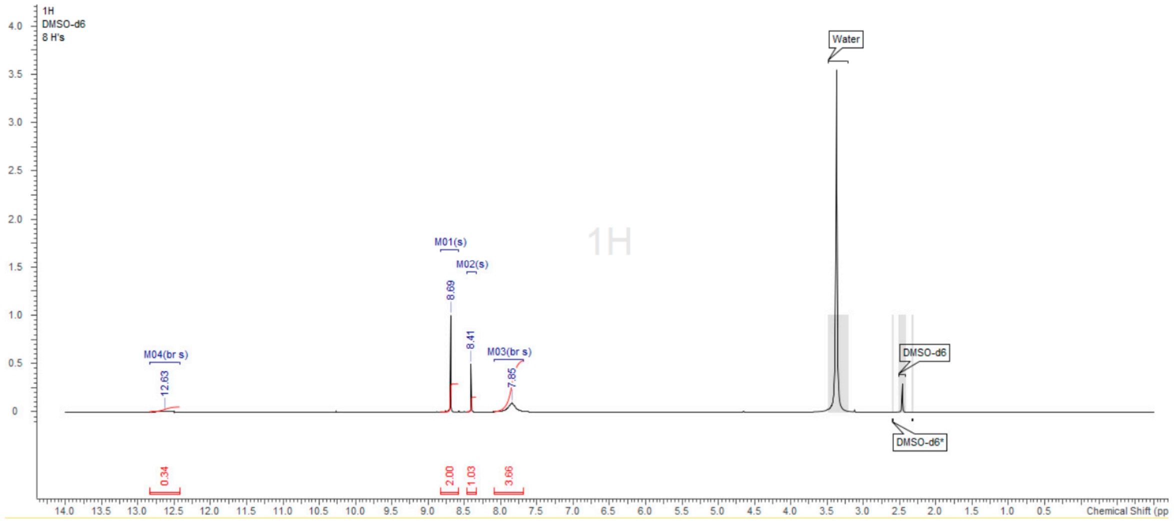

Figure S30. ^{1}H NMR (500 MHz, $(CD_3)_2SO$) spectrum of compound JP14

^{13}C NMR (126 MHz, DMSO-*d*$_6$): δ 155.89, 148.93 (2C), 140.82, 136.91, 131.01 (2C)

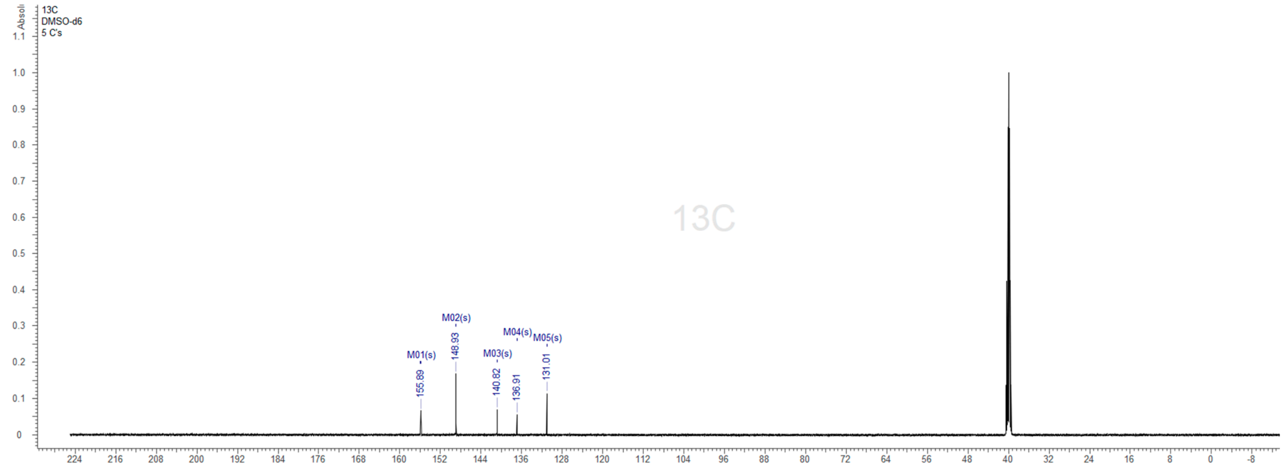

Figure S31. ^{13}C NMR (126 MHz, $(CD_3)_2SO$) spectrum of compound JP14

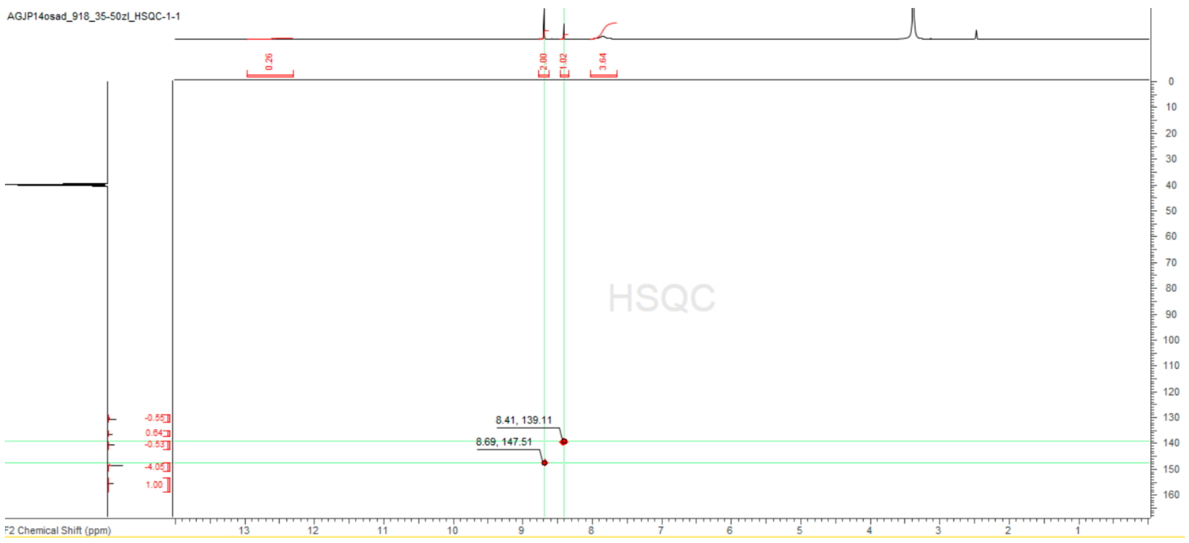

Figure S32. HSQC-NMR spectrum of compound JP14

MS (ESI$^+$) m/z: 233.83 [M + H$^+$]

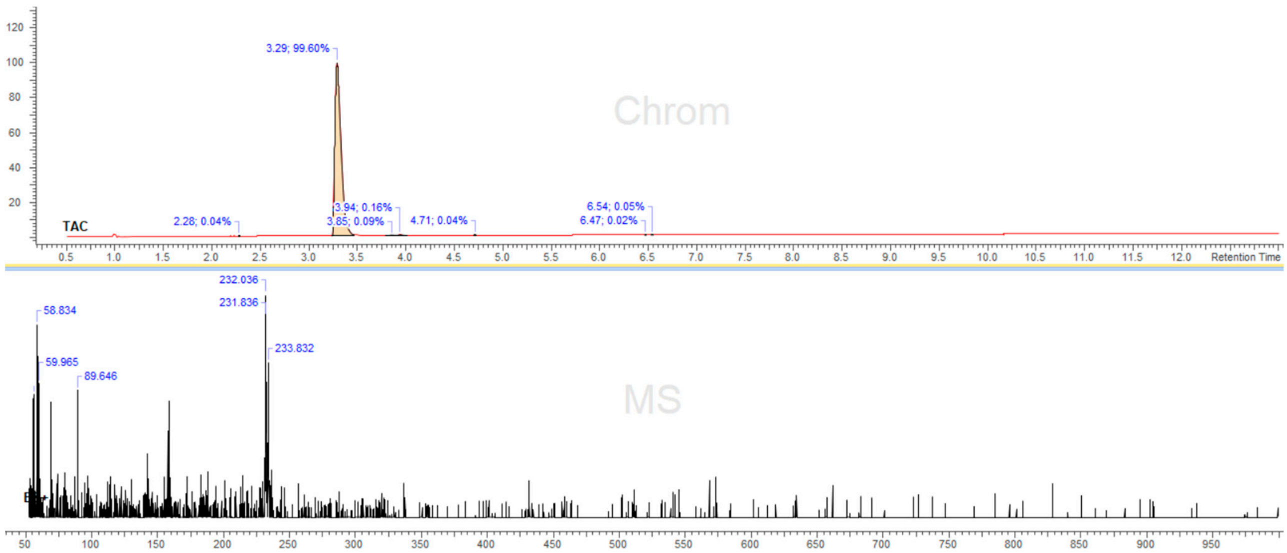

Figure S33. LC-MS chromatograms of compound JP14 (99.60%)
